# Professionals’ Awareness of Young Carers in Schools: Results from a French Survey

**DOI:** 10.3390/ijerph192114172

**Published:** 2022-10-29

**Authors:** Aurélie Untas, Christel Vioulac, Pauline Justin, Agnes Leu, Géraldine Dorard

**Affiliations:** 1 Laboratoire de Psychopathologie et Processus de Santé, Université Paris Cité, F-92100 Boulogne Billancourt, France; 2 Institute for Biomedical Ethics, University of Basel, Bernoullistrasse 28, 4056 Basel, Switzerland

**Keywords:** awareness, caring, identification, occupation, professionals, school, support, young carers

## Abstract

Few studies have investigated professionals’ awareness of young carers (YCs). Therefore, the main aim of this study was to explore school professionals’ awareness of YCs. The secondary aim was to compare professionals’ responses according to their occupation. A total of 2658 professionals took part in this study by completing an online questionnaire. The results indicated that the mean for the perceived age for care beginning was 10.3 years old, with parents being perceived as the most frequent care recipient. The main caring activities reported were domestic chores, emotional support and sibling care, while care provided directly to the care recipient was perceived as less frequent. Many suggestions for improving the identification, referral and support of YCs were seen as relevant, but professionals were less supportive of suggestions for adjustments within schools. The key training needs centered around better identifying YCs and developing better knowledge of their difficulties. Differences were observed according to occupation, with administrative staff and teachers having less accurate perceptions than school counsellors as well as social and health professionals. This study shows that it can be difficult for school professionals to imagine a child providing care. Specific training, therefore, needs to be proposed according to school professionals’ occupations.

## 1. Introduction

When children and adolescents are confronted with the illness or disability of a family member, they may have to take on a caring role. Those who take on such a role are called young carers (YCs). According to Becker [1], YCs are “children and young persons under 18 who provide or intend to provide care, assistance or support to another family member. They carry out significant or substantial caring tasks, and often regularly assume a level of responsibility usually associated with an adult. The person receiving care is often a parent but can be a sibling, grandparent or another relative who is disabled, has some chronic illness, mental health problem or other condition connected with a need for care, support or supervision” (p. 378). YCs take on a wide range of tasks. Some tasks are comparable to those that a typical youth might provide, such as household tasks, sibling care or administrative and financial support [2,3], but they are provided more frequently. Other responsibilities are specifically related to the care recipient’s health situation, such as providing emotional, general, and nursing-type care. Caregiving could begin as young as the age of five, although it more commonly begins between the ages of 10 and 15 [2,3].

Numerous studies have investigated the consequences of being a YC. They showed that assuming such a role can have positive and negative consequences on a physical, psychological, social or educational level [3,4,5,6,7,8]. Specifically, regarding education, some youths experience academic difficulties, including problems concentrating in class, failure to complete homework, absenteeism, discrimination, and school dropout [2,3]. These factors can potentially affect long-term life plans [9]. Despite these difficulties, youths note that school is a place in which they can withdraw from their caring activities and concentrate on other aspects of their lives [7,10,11]. However, it has been shown that YCs would like school professionals to be more aware of their family situations and to provide more support [2,7,12]. Therefore, school professionals may play a significant role in identifying and supporting YCs [13,14,15].

Only a few studies have thus far investigated school professionals’ perceptions and experiences of YCs. In the United Kingdom, a recent qualitative study was conducted among school teachers [16]. This country is the most advanced regarding YCs and results showed that all respondents had significant experience in supporting YCs [17]. However, they perceived many difficulties identifying YCs who did not want to openly share any information about their situation. The perceived main enabler of identification was the trust relationships between the school, the pupil, and the parents. Studies in the United Kingdom have shown that schools played an extended role in supporting families through the pandemic, especially vulnerable families [18,19], underlying the particular place of schools in children’s and family lives. 

Two Swiss studies investigated professionals’ awareness of YCs [14,20] by looking at healthcare, education, and social services professionals. The first study was qualitative [14] and showed that professionals had a low level of awareness of the issue of YCs but a willingness to engage with the subject. Professionals also expressed an interest in accessing tools to help them support YCs. The second Swiss study was a national survey [20] which showed that professionals from the field of education considered the topic of YCs to be not particularly relevant to their occupation and therefore felt less able to identify and support YCs than those from the fields of healthcare and social work. Yet, this study did not investigate whether, within the field of education, there were differences according to professionals’ specific occupations (e.g., teachers vs. school counsellors or school nurses). In France, a qualitative study showed that most school professionals were not aware of the term “young carer” even though they regularly met YCs in their practice [13]. Although they did not know how to identify and support YCs, they were at least able to provide some perceptions of their difficulties. Some specificities according to professionals’ occupations showed that social and healthcare professionals and school counsellors were more aware of YCs than other professionals (e.g., teachers). This may be because these professionals are more likely to share intimate information with youths experiencing difficulties. Therefore, they may be more familiar with the family context and thus with young people facing the illness or disability of a relative. Those specificities according to professionals’ occupations need to be better investigated because they might affect professionals’ awareness. They should also provide insights necessary to improve the training of professionals according to their occupation.

In the literature, the prevalence of YCs is relatively varied across countries. It ranges from 3.2% to 22% depending on the criteria and methodology used to identify YCs and the age range studied [21,22,23,24]. Moreover, many complexities exist in identifying YCs, including cultural barriers, stigma, and self-identification difficulties. In France, the only data available relates to high school students (predominantly aged 15–17) and shows that 14.3% could be considered adolescent YCs [25], which amounts to as many as 294,600 adolescent YCs. Unfortunately, the proportion of YCs under the age of 15 in France is unknown, but it is conceivable that the overall number of YCs is much higher when all age groups are considered.

Even if all YCs may not need help, it is still important to better understand the level of school professionals’ awareness of them. In France, recognition of, research into, and support for YCs is recent and much work remains to be done to increase awareness and improve policy responses [17].

Hence, this study aimed to explore school professionals’ awareness of YCs in a large sample. More specifically, the purpose was to investigate: (1) their perceptions of YC characteristics (age, care recipient) and their caring activities; (2) their perceptions of the consequences of this situation for YCs regarding general negative and positive consequences as well as those specific to school; and (3) their perception of what could be done in schools to better identify and support YCs, associated barriers, and needs for specific training. The aim was to explore these aspects among general school professionals and compare professionals according to their specific occupations within education. In line with the results of a qualitative study by Justin et al. [13], we expected that social and healthcare professionals, as well as school counsellors would have more accurate perceptions than teachers, principals, and other professionals.

## 2. Materials and Methods

### 2.1. Participants

The sample comprised a range of school professionals in France. Participants were included if they were working with children and adolescents aged six to 18 in French schools (primary, secondary, or high school). Further inclusion criteria related to whether they were working at least part-time (i.e., 17.5 h per week in France), had more than one year of professional working experience, had provided consent to participate in the study, and had completed the questionnaire entirely. A total of 4261 participants agreed to participate in the study, from which 2658 professionals completed the questionnaire.

The participants’ mean age was 45.2 years (SD = 9.9,) and 81.8% were female. The majority of participants were teachers (40.5%), but many others took part: principals/vice principals (18.1%), school nurses (10.0%), psychologists (7.1%), school social workers (5.3%), school counsellors (4.9%), school doctors (1.6%), and others (4.0%). Most worked in public schools (89.5%), while a minority worked in educational priority schools (i.e., schools having the financial support to decrease social inequalities because pupils have more social difficulties in those areas; 22.2%). They had on average, 17.87 years of experience working in education (SD = 10.4). More than half of the participants worked in secondary or high schools with pupils aged 11–18 (58.6%), 34.4% in elementary schools (children aged 6–10), and 7% in both types of schools.

### 2.2. Procedure

An online survey was conducted. Professionals were informed about the study primarily through an online-accessible national database of French schools, which provides email contacts for each school. Through these contacts, principals were asked to participate in the study and inform their fellow school professionals about it. Some professionals were also directly contacted if their contact details were accessible on their school websites, particularly school psychologists and health and social care professionals. Social media was also used to disseminate the call for participation. Invitations to participate in the study provided links to a short video introducing the study, an information notice, and a link to access the online survey. The survey was anonymous, took approximately 20 min to complete, and was accessible online from March 2020 to February 2021.

### 2.3. Measures

The study questionnaire was based on the results of a qualitative study undertaken among French school professionals [13] and a comprehensive literature review. This choice was made to explore each theme in-depth. It was composed of five sections: participants’ characteristics, knowledge and experience of YCs, perceived activities and consequences of being a YC, professionals’ needs related to YCs at school, and personal information about the caring role of the participants. This paper presents the results regarding the perceived activities and consequences of being a YC and professionals’ needs related to YCs at school.

*Participants’ characteristics.* Several questions assessed sociodemographic information (gender, age, living status) and professional information (profession, work experience, type and age level of school, and department).

*Perceived activities and consequences of being a YC.* This section was composed of five parts. The first two pertained to possible negative and positive consequences of being a YC. The first part listed eight school consequences (e.g., behavior or concentration issues during class, absenteeism, and overinvestment). The second part listed 14 general consequences (e.g., sadness, fatigue, maturity). For these two parts, responses were given according to a five-point Likert scale (from 1—never to 5—very often). Participants could additionally choose the response “I do not know”. In the third part, participants were asked who the three most frequent care recipients were. The following options were listed: classmate, friend, parent, sibling, grandparent, other family member, neighbour, or other person. The fourth section listed 11 activities in which a YC could help (e.g., domestic chores, care of siblings, medical care). Responses were as follows: ‘never’, ‘once a month or more’, ‘once a week or more’, ‘almost every day’, and ‘I do not know’. The final part asked at which age children start becoming YCs. According to the literature, responses were considered relevant from age five. In addition, professionals were asked why, in their opinion, YCs did not talk more about their situation at school. Again, professionals chose from a list (e.g., ‘they do not know they are a YC’, ‘they are afraid to be stigmatized’, ‘they think the school is not interested in their family situation’).

*Professionals’ needs regarding YCs at school.* Sixteen suggestions to better identify and support YCs were listed. These were then grouped into four sections: enhance identification of YCs at school (three suggestions), school arrangements (five suggestions), helping and/or referring the youth and the family (five suggestions), and raising awareness among youth (three suggestions). For each, participants had to indicate whether they felt it was relevant by answering, “yes”, “rather yes”, “no”, or “I do not know”. Participants were next asked to indicate the main obstacles to implementing the suggested strategies (e.g., lack of time, training, and financial/staff support). Finally, a list of training themes was proposed, and participants were asked which they would be interested in (e.g., developing better knowledge of YCs and the difficulties they face, being able to better identify YCs, and having techniques to talk with YCs about their situation).

### 2.4. Data Analyses

Descriptive analyses were performed on the sample. Some responses were clustered to simplify the presentation of the results: (1) For activities in which a YC could help; responses were categorized as “never or occasional” (combination of “never” and “once a month or more”), “frequent” (combination of “once a week or more” and “almost every day”), and “do not know”; (2) For school and general consequences of being a YC, responses were categorized as “never to sometimes” (Likert response 1-never to 3-sometimes), “often or very often” (Likert response 4-often and 5-very often), and “do not know”; (3) For suggestions to better identify and support YC, responses were categorized as “relevant” (combination of “yes” and “rather yes”), “not relevant”, and “do not know”.

Group comparisons were undertaken by occupation using a chi-square test of independence to investigate associations between those responses and professional occupation. Four groups were compared: “administrative staff”, which was composed of principals/vice principals and other professionals; “teachers”; “counsellors”, which was composed of school counsellors and school monitors; and “social and healthcare”, which was composed of school social workers, psychologists, nurses, and doctors. The null hypothesis was that no relationship exists between professionals’ responses (i.e., perceived activities of YCs, consequences of being a YC and professionals’ needs regarding YCs at school) and occupation. Data analyses were conducted using IBM SPSS Statistics (version 25.0; SPSS Inc., Chicago, IL, USA).

## 3. Results

### 3.1. Perception of the Age at Which Caring Begins

Regarding the age at which a child can become a YC, the mean age reported was 10.3 years old (SD = 2.98). Most participants stated an age between six and 10 (53.8%), followed by an age between 11 and 15 (31.3%), then between 16 and 22 (10.5%), and finally, a few under six (4.4%).

There was a significant difference according to occupation (Welch = 19.006; *p* < 0.001), with school counsellors reporting a higher age than all the other professionals (11.67 vs. 10.42 for administrative staff, 10.08 for teachers and 10.51 for social and healthcare professionals).

### 3.2. Perception of the Person a YC Cares for

When asked about the three main persons that a YC might care for, professionals’ most frequent answer was a parent (65.3%), followed by a sibling (56.5%), and then a grandparent (34.4%).

Comparisons by occupation showed that social and healthcare professionals were more likely to choose a parent as the first person (78.2%), whereas administrative staff and teachers were more likely to choose a sibling (respectively, 24.3% and 25.4%; χ^2^ = 90.342; *p* < 0.001).

### 3.3. Perceived Caring Activities

Regarding general help, the activities perceived as the most frequently provided by YCs were as follows: taking daily care of brothers and sisters (83.3%), helping with domestic chores (82.4%), and having responsibilities toward brothers and sisters (76.5%). Help in administrative tasks was much less frequent (36.7%), and providing financial support was infrequent (9%). For these two last activities, a much more significant proportion of participants stated that they were unaware of whether YCs provided this support.

Regarding the care recipient, the three activities perceived as the most frequent were the following: helping with mobility and bringing things to the care recipient (77.2%), providing emotional support (73.7%), and being present to ensure that everything is fine for the care recipient (68.2%). Activities such as helping the care recipient with daily activities (58.0%), providing medical care (41.3%), and illness management (32.4%) were less frequent, and many professionals stated that they did not know whether YCs provided this kind of support (see Table 1).

**Figure 1:**
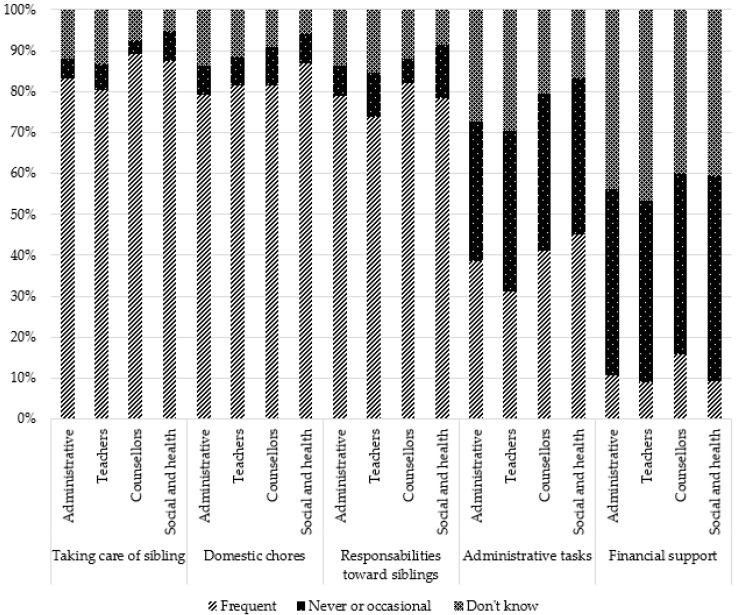
Perception of general help provided by young carers according to occupation.

**Figure 2:**
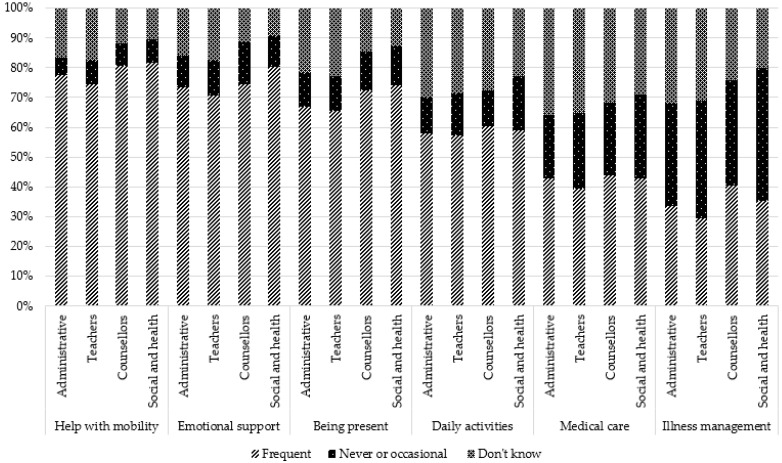
Perception of help provided to the cared-for relative by young carers according to occupation.

Comparisons by occupation showed significant differences for each type of support. Administrative staff and teachers more often responded “do not know” than counsellors and social and healthcare professionals did. Social and healthcare professionals reported more than other professionals that YCs frequently helped with domestic chores, taking daily care of siblings, administrative tasks, mobility/bringing things, emotional support, and being present to ensure that everything was fine for the care recipient. They also reported more than other professionals that responsibilities toward siblings, helping the care recipient in daily activities, and providing medical care occurred frequently. Counsellors reported more that YCs frequently helped in taking daily care of siblings, offering financial support, and illness management, while these types of support were less reported by social and healthcare professionals. These results are presented in Table 1 and Figure 1 and Figure 2.

### 3.4. Perceived Consequences of Being a YC

Regarding general consequences, participants perceived that the most frequent negative consequences were sadness (81.2%), worries (66.2%), and fatigue (65.0%). Other negative consequences were perceived as being much less frequent: isolation (24.8%), physical problems (20.8%), anger (20.8%), shame (19.7%), dark thoughts (10.9%), and at-risk behaviors (7.4%). For the two last consequences, the proportion of participants stating that they “do not know” whether these consequences exist for YCs is relatively large (26.3% and 36.3%).

Positive consequences were perceived in a large proportion: maturity (81.2%), autonomy (72.3%), altruism (56.8), and in a lesser proportion, personal enrichment (22.0%) and self-confidence (20.8%).

Regarding more specifically school-based consequences, the most frequent were concentration issues (64.1%), school difficulties (49.5%), absenteeism and lateness (48.9%), and impact on educational progress, including choices regarding future education (46.8%). A lesser proportion was reported repeating a class (24.6%) or dropout (24.6%), having behavioral issues in class (24.3%), and school overinvestment (18.1%). Stigmatization or harassment was not perceived as frequent (6.2%).

Comparisons by occupation showed that teachers reported more frequently that they “do not know”, regardless of the type of consequence. Administrative staff more frequently reported “never to sometimes” for some negative consequences (sadness, anxiety, fatigue, aggressivity) and consequences related to school (behavior, concentration, absenteeism, stigmatization). However, for two positive consequences (self-confidence and personal enrichment), they reported more frequently “often–very often”. Social and healthcare professionals reported more frequently “often–very often” for all general negative consequences, for one positive consequence (maturity), and school consequences (except overinvestment and stigmatization). They reported more frequently “never to sometimes” for positive consequences (self-confidence, personal enrichment, altruism/empathy). Counsellors reported more frequently “often–very often” for three school consequences (concentration issues, absenteeism, and repeating a class) and more frequently “never to sometimes” for three negative consequences (anxiety, physical health, and addictive behaviors) and one positive consequence (personal enrichment).

**Figure 3:**
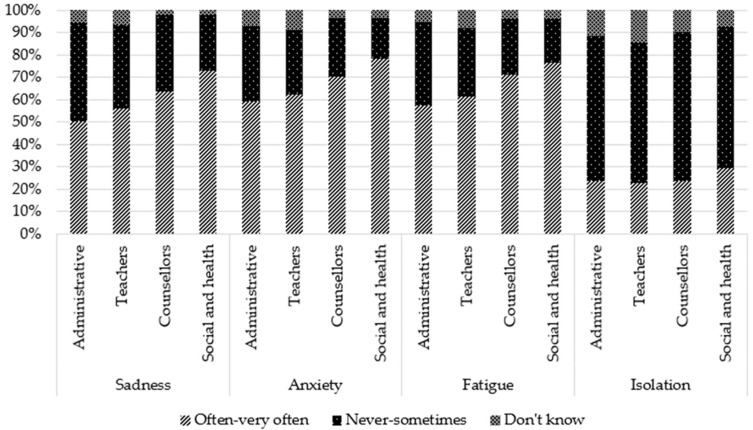
Perception of general negative consequences according to occupation: sadness, anxiety, fatigue, isolation.

**Figure 4:**
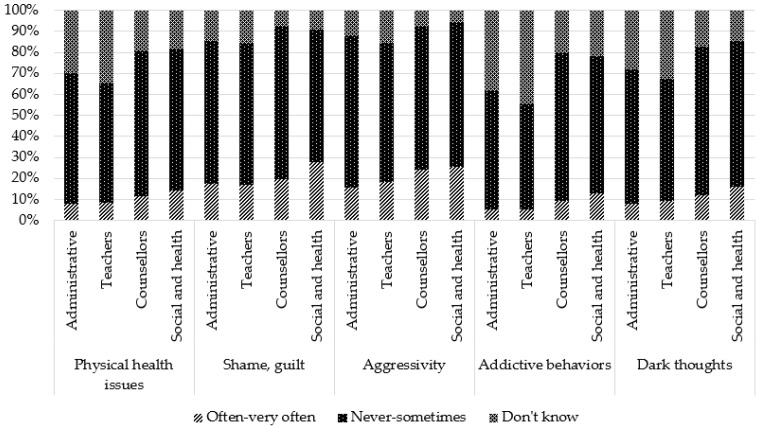
Perception of general negative consequences according to occupation: physical health issues, shame, aggressivity, substance abuse, dark thoughts.

**Figure 5:**
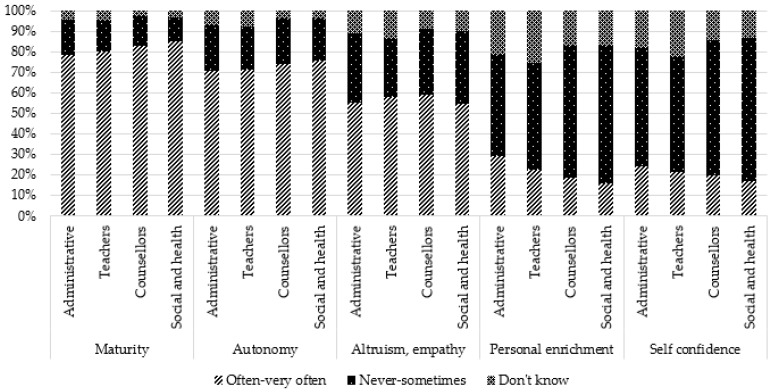
Perception of general positive consequences according to occupation.

**Figure 6:**
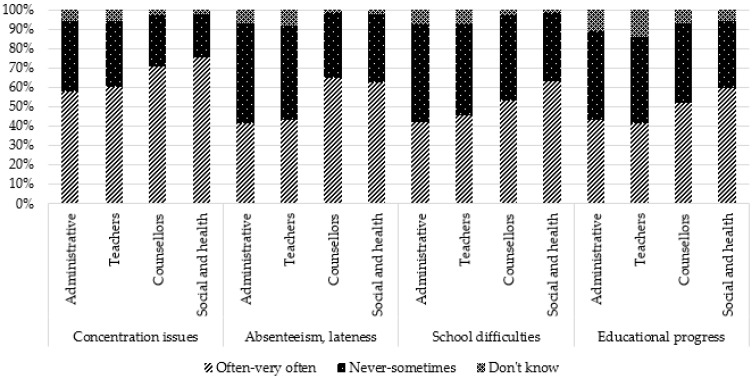
Perception of school consequences according to occupation: concentration issues, absenteeism/lateness, school difficulties, educational progress.

**Figure 7:**
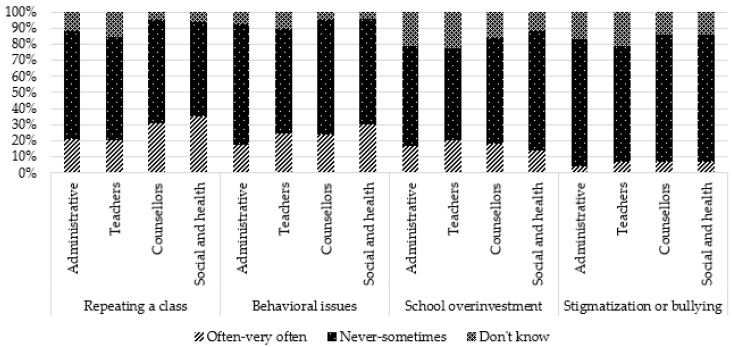
Perception of school consequences according to occupation: repeating a class, behavioral issues, school overinvestment, stigmatization or bullying.

These results are presented in Table 2 and Figure 3, Figure 4, Figure 5, Figure 6 and Figure 7.

### 3.5. Perceived Reasons Why YCs Do Not Talk about Their Situation at School

The two main reasons suggested by the professionals as to why YCs do not talk about their situation at school were “because they are not aware they are a YC” (77.8%) and “because they do not want the situation to be known at school” (70.5%). The other reasons were “because they are afraid to be separated from their family” (51.3%), “because they think it’s pointless” (45.4%), “because they think school is not interested in their family situation” (45.1%), “because they are afraid to be stigmatized” (27.7%), and “because they are afraid not to be understood by the school staff” (25.5%).

Regarding comparisons by occupation, school counsellors and social and healthcare professionals more frequently reported the following reasons: “because they are afraid to be separated from their family” (χ^2^ = 74.050; *p* < 0.001), “because they think it’s pointless” (χ^2^ = 21.074; *p* < 0.001), and “because they are afraid not to be understood by the school staff” (χ^2^ = 40.436; *p* < 0.001). Social and healthcare professionals also reported more frequently “because they do not want the situation to be known at school” (χ^2^ = 19.097; *p* < 0.001) and “because they think school is not interested in their family situation” (χ^2^ = 11.333; *p* = 0.010).

### 3.6. Suggestions to Better Identify and Support YCs

Most suggestions to better identify and support YCs were perceived as relevant. Those favoring a better identification of YCs at school, raising schoolchildren’s awareness of the situation of YCs, and helping and/or referring the youth and the family were all largely perceived as relevant, with the percentage varying between 84.2% (“collecting information from the youth and the family”, “providing information at school about disabilities, illness and help provided in the family”) and 98.7% (“actively listening to the YC”). Only “taking contact with social services” was perceived as less relevant (66%). Regarding academic accommodations, if “set-up mentoring or personalized homework support” (88%), “adapting deadlines to deliver personal work” (76.9%), and “creating monitoring committee for pupils/YC referent” (69.9%) were globally perceived as relevant, “accommodation of time schedule” (51.2%) and “authorizing phone use at school to be able to make contact with the care recipient” (36.9%) were perceived as being much less relevant.

For several suggestions, no significant difference was observed according to occupation. However, caution should be exercised when interpreting these results because the chi-square test of independence conditions was violated (i.e., there were less than five participants for some response modalities). However, school counsellors more frequently reported that suggestions related to school arrangements, such as the adaptation of deadlines and time schedules, as well as personalized tutoring, were not relevant. Teachers more frequently reported follow-up committees as being relevant but said they “do not know” in regard to time schedules. Administrative staff more frequently perceived adaptation of work deadlines and making contact with social services as relevant but saw follow-up committees and making contact with families as not relevant. Social and healthcare professionals, meanwhile, more frequently reported suggestions related to helping and/or referring the YC and the family (e.g., encouraging contact between the youth and social and healthcare professionals, making contact with the family and with social services) as relevant, whereas teachers reported them more frequently as not being relevant or they said they “do not know”.

**Figure 8:**
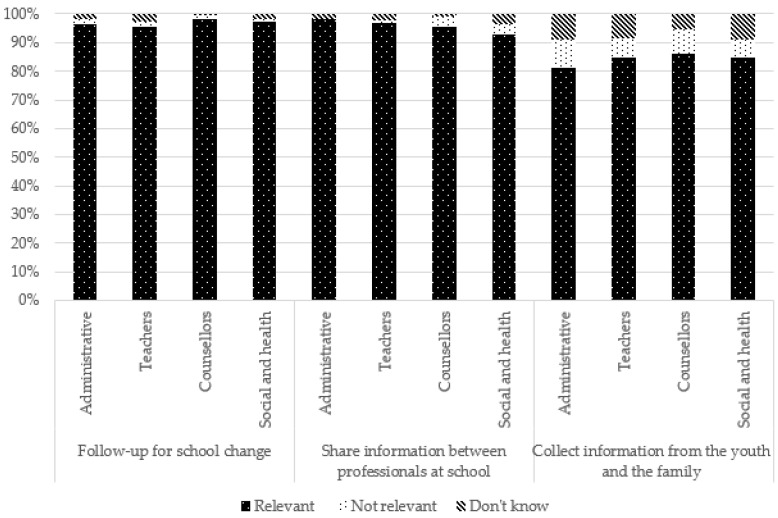
Suggestions to enhance identification of young carers at school according to occupation.

**Figure 9:**
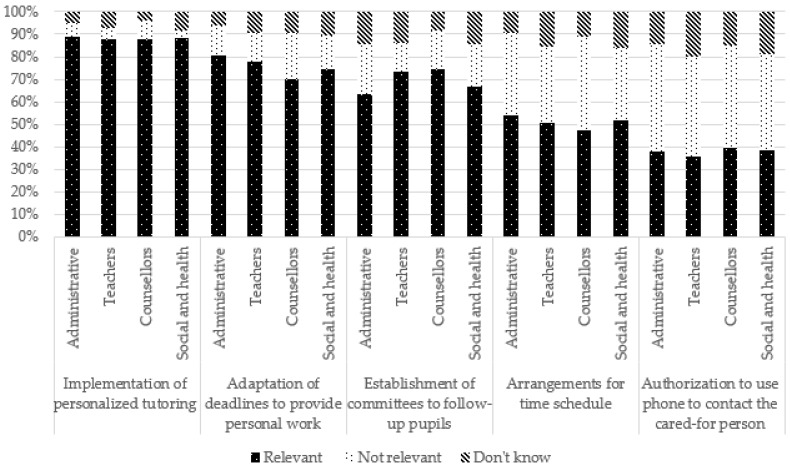
Suggestions for school arrangements according to occupation.

**Figure 10:**
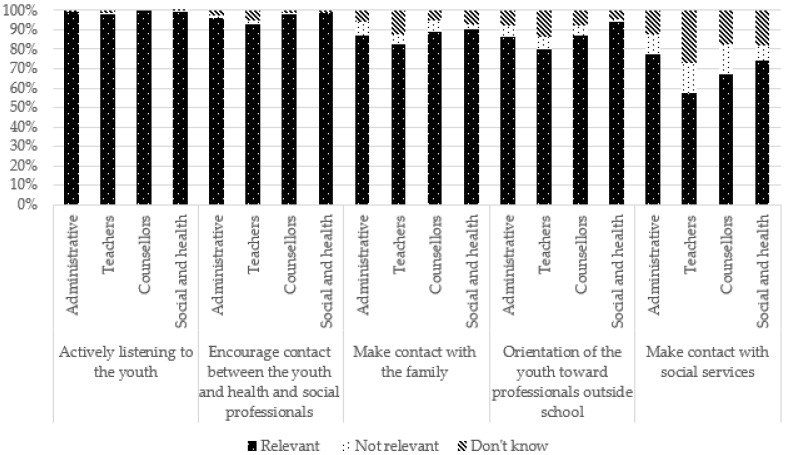
Suggestions to help and/or refer the youth and the family according to occupation.

**Figure 11:**
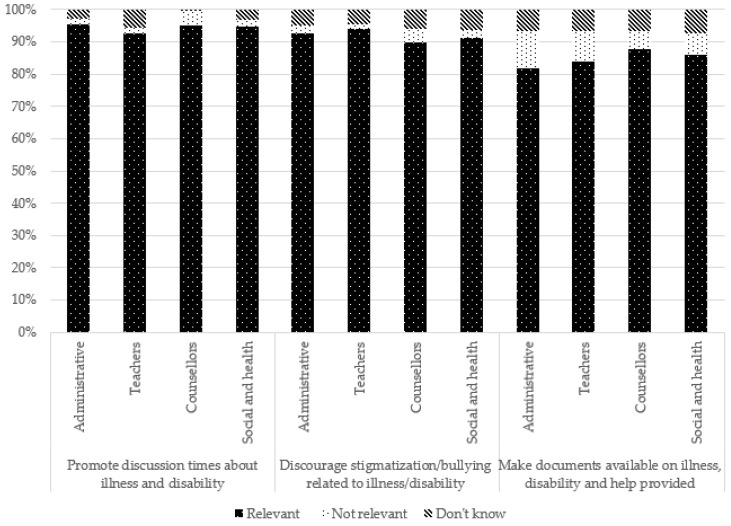
Suggestions to raise awareness in schools according to occupation.

These results are presented in Table 3 and Figure 8, Figure 9, Figure 10 and Figure 11.

### 3.7. Perceived Obstacles to Identify and Support YCs

Perceived obstacles to identifying and supporting YCs were mostly lack of time (65.6%), lack of training (62.9%), and lack of financial/staff support (59.9%), and less frequently the limitations of their occupational role (9%) and the fact that it was not the school’s role to become involved in such matters (3.2%).

Teachers reported more frequently lack of training (χ^2^ = 106.629; *p* < 0.001), lack of financial/staff support (χ^2^ = 23.740; *p* < 0.001), and it not being relevant/related to their occupational role (χ^2^ = 69.507; *p* < 0.001).

### 3.8. Themes for Which Professionals Would Like Training

Themes for which professionals would like training were as follows: better identifying YCs (59.4%), developing a better understanding of YCs’ difficulties (51.4%), being counselled about the best way to approach YCs and their families (44%), learning techniques to communicate with the YC about their situation (36.9%), developing knowledge of academic accommodations for YCs (36.2%), understanding educational guidance opportunities for YCs (26.7%), and providing follow-ups on YCs (25.8%). Six percent of the participants indicated they were not interested in any of these themes.

School counsellors and social and healthcare professionals reported more frequently that they would like training to help them better identify YCs (χ^2^ = 14.336; *p* = 0.002) and to develop knowledge of educational guidance opportunities for YCs (χ^2^ = 114.798; *p* < 0.001). Teachers, by contrast, reported more frequently that they would like training to develop techniques to communicate with the youth about the situation (χ^2^ = 26.213; *p* < 0.001) and to be counselled about the best way to approach YCs and their families (χ^2^ = 31.170; *p* < 0.001).

## 4. Discussion

This study shows that school professionals have perceptions about YCs’ characteristics and caring activities that are in line with those reported in the literature among youth. Many suggestions to enhance the identification and support of YCs were perceived as relevant, although those related to specific school arrangements were perceived as less relevant. Training needs that were identified consisted primarily of better identifying YCs and better understanding their difficulties. If previous studies have emphasized the low level of professionals’ awareness of YCs, this research clarifies their perceptions within a large sample of professionals at schools and their specific perceptions according to their occupation. This is a major point as each type of school professional may have a key role to play in the lives of YCs, with particularities depending on their role and skills.

### 4.1. Professionals’ Awareness of Young Carers

This study showed that professionals reported that youths could become YCs at around 10 years old, with more than half of the professionals reporting ages between six and 10 and approximately a third reporting ages between 11 and 15. This suggests a relatively accurate perception because, even if caring can technically begin as young as the age of five, it does, in fact, become more important for young people aged 10 to 15 [2,3] due to several factors, such as child development, parenting and attachment [26]. 

Regarding the care recipient, in our study, professionals reported parents as the most likely recipient of care, siblings as the second most likely, and grandparents as the third most likely. These results are in line with several studies in the literature among youth, which showed that YCs most often care for an ill or disabled parent [23,24,25,27]. However, those studies observed that the proportion of grandparents was higher than the proportion of siblings. Our result could be explained by the fact that professionals more easily imagine children caring for a relative at home, which is less often the case for grandparents. 

YCs might provide support for a wide range of tasks, the main ones generally being domestic chores as well as household and emotional support [22,23,25,28]. Professionals did report those types of support more frequently, but they also mentioned sibling care, which is a little less reported in studies among youth. Caring activities related to the care recipient (daily activities such as eating or washing, medical care, and illness management) were much less reported as frequent and around a third of the professionals stated that they did not know whether YCs provided this kind of support. This shows how it might be difficult for professionals to imagine a child playing the role of carer [29]. This may also reflect YCs’ reluctance to discuss caring matters openly.

The results showed that sadness, anxiety, and fatigue were the potential negative consequences of being a YC that were the most reported, while all others were much less reported. Those consequences are the most often reported in the literature [4]. This may be because they are easier to imagine or to observe and are probably the most observed among pupils at school regardless of their family situation. Indeed, professionals in France are trained to identify those warning signs related to children’s well-being [30]. When professionals identify those signs, the question is whether they can connect them with a potential situation of a YC. 

Regarding potential positive consequences, maturity and autonomy were the most frequently reported, whereas empathy, personal enrichment, and self-confidence were less reported. Those have also been observed in the literature, but they mainly develop when the care provided by the YC is recognized [29,31].

In terms of school-related consequences, concentration issues were the most commonly reported. Meanwhile, school difficulties, absenteeism or lateness, and impact on educational progress were reported less frequently. Repeating a class, behavioral issues, and school overinvestment were even less reported, and stigmatization or bullying were perceived as being very rare. These results are in line with those observed among YCs [32].

The suggestions to better identify and support YCs were largely perceived as relevant by professionals. Those perceived as the most relevant (for more than 95% of the professionals) were as follows: actively listening to the youth, ensuring a better follow-up for school change (e.g., the transition from primary to secondary school), sharing information between professionals, and encouraging contact between the youth and health and social professionals at school. These activities are already undertaken in schools for other issues and may be easier to undertake for professionals. It is very positive that they are perceived by professionals as applying to YCs.

Other suggestions were also perceived as very relevant (84% to 88% of the professionals): implementation of personalized tutoring or help with homework, making contact with the family to discuss the situation, referring the youth to professionals outside school, and collecting information from the youth and the family. Those actions are also already undertaken in schools but are more specific to some occupations. As Fasciano et al. [33] pointed out, professionals can face difficulties dealing with the topic of disease with the children and their parents because of fear or lack of knowledge regarding it. This can also explain the results regarding the suggestions for raising awareness (i.e., promoting discussions among youths regarding illness and disability, discouraging stigmatization/bullying, and making documents on YCs available), which was perceived as being relevant in a relatively similar range.

More personalized suggestions, such as adaptation of deadlines to provide personal work, the establishment of committees to follow up on pupils (or having a school career referent for YCs), and arrangements for time schedules, were perceived as the least relevant (51% to 77% of the professionals). Such arrangements as these require professionals to agree that YCs may have specific needs regarding their education. This finding can be linked to that of Justin et al. [13], who observed that some school professionals feared that YCs could take advantage of this kind of support or create misunderstandings among other pupils. This result also questions whether professionals perceive being a YC as a temporary situation, not a long-term one, suggesting that YCs were less in need of specific adaptations at school. Unfortunately, this aspect was not investigated in our survey.

School professionals perceived the main obstacles to identifying and supporting YCs were lack of time, training, and financial/staff support. This result regarding time and staff support may be even more important because of the context of the COVID-19 crisis, which has applied a significant amount of pressure on school professionals [34]. Moreover, there is currently a huge problem in France with many school professionals having left their occupations and schools failing to employ a sufficient number of staff (Letter of the Minister of Education, Pap N’Diaye, to the teachers (27 June 2022): https://www.education.gouv.fr/lettre-de-pap-ndiaye-aux-professeurs-341884 (accessed on 20 July 2022)). Regarding the lack of training, this is in line with the needs that professionals reported, which were mainly related to better identifying YCs, better understanding the YC topic, and being counselled about the best way to approach YCs and their families.

### 4.2. Specificities According to Professionals’ Occupation

Differences observed among professionals according to their occupation confirm the results observed in Justin et al.’s [13] qualitative study. Social and healthcare professionals seemed to have the highest awareness, followed by school counsellors, and, finally, teachers and administrative staff. Indeed, social and health professionals and school counsellors reported more diversity in caring activities, especially those related to the care recipient. Social and health professionals were also those who most frequently reported potential negative consequences (on a general level) and less positive consequences for personal enrichment and self-confidence. In line with the specificities of their occupation, they were those who perceived the suggestions related to helping and/or referring to the youth and the family as the most relevant. They were also, alongside school counsellors, the most interested in training related to better identifying YCs and understanding educational guidance.

Regarding the administrative staff and teachers, they perceived caring activities or their consequences as less frequent or selected the “do not know” response. Teachers reported more obstacles but also more frequently identified the need for training related to communication with the youth and the best way to approach their families. These results could be explained by the fact that social and health professionals as well as school counsellors are more likely to share family and intimate information with youths having difficulties and thus may be more aware of YCs, whereas teachers and administrative staff might be less aware. This has to be considered for the development of specific training for these professionals and the role they might play in YCs’ lives at school. Teachers are the professionals who spend the most time in contact with pupils and can therefore best observe pupils’ difficulties and thus identify potential YCs. Indeed, in the United Kingdom, YCs at primary school are most frequently identified by teachers and support staff [35].

### 4.3. Practical Implications

The results of this study suggest several practice implications. Firstly, the results underline the need for a significant campaign among school professionals to enhance awareness of YCs. Given the relatively accurate perceptions shown in this study, the primary purpose of these campaigns would be to highlight the situation of YCs and to provide the vocabulary to accurately describe their situations. Secondly, short training sessions could be provided to professionals to help them better understand the specificities of YCs (characteristics, caring activities, consequences, and how school might help them). Such training could be provided to all professionals. Thirdly, training could be specific according to occupation. For social and health professionals and school counsellors, training about strategies and specific tools to better identify YCs and educational guidance could be proposed. Training to enhance their self-efficacy in communicating with the youth and their families could be proposed for teachers. This would probably help them to feel more at ease regarding those situations and to better identify YCs and refer them to the relevant social or health professional at school. Fourthly, each school should be encouraged to discuss the topic of YCs among the school staff to determine strategies to better identify and support YCs. For example, specific measures could be undertaken to enhance awareness of YCs among pupils (e.g., meetings, flyers). All these suggestions would benefit from the support of the French Ministry of Education to encourage schools to engage themselves in these kinds of actions. Unfortunately, this is still not the case. However, some departments have begun to mobilize themselves. Other countries’ experiences, such as that of the United Kingdom, are very important to consider, e.g., regarding the use of a guide proposal [36,37].

### 4.4. Limitations

It should also be noted that this study has several limitations. The first relates to the sample. Even if it is relatively large, it cannot be considered entirely representative of school staff in France. Indeed, nearly every school in France received an email about the study, but each principal was personally responsible for disseminating the survey inside their respective schools. This dissemination could have benefited from the French Ministry of Education’s support but it was completely overwhelmed by the COVID-19 crisis. Another limitation is that professionals who took part in the study were probably more concerned about pupils having a relative with an illness/disability, which might have influenced the results in a positive way. A third limit is related to the questions we asked, particularly those related to the consequences of caring. We decided to investigate various consequences based on the qualitative study [13] and a literature review. As some items referred to observable effects (i.e., self-harm) and others did not (i.e., dark thoughts), professionals may have had difficulties answering, leading to speculation. Finally, we can ask to what extent social desirability influenced participants’ responses, especially regarding suggestions to better support and identify YCs, as those suggestions were largely perceived as being relevant. Moreover, the suggestions perceived as the most relevant were those already undertaken by professionals at school for other issues.

## 5. Conclusions

This study is the first in France to cover a large sample of school professionals. It shows that professionals’ awareness of YCs has to be enhanced in schools. Moreover, a deeper reflection has to be undertaken to support YCs throughout their education.

Further research should develop training for school professionals and evaluate their impact. It should also explore professionals’ experience with YCs, once trained about the topic, to understand the difficulties and obstacles they might face in identifying and supporting YCs as well as the resources they may require facilitating such efforts.

## Figures and Tables

**Table 1:** Perceived activities in which YCs could help.

	Entire Sample			Comparison by Occupation	
	Frequent	Never or Occasional	Do Not Know	χ^2^	*p*
**General help**					
Taking daily care of siblings (taking them to school, to activities …)	83.3%	6.0%	10.8%	34.871	<0.001
Helping in domestic chores (storing, cleaning, cooking, shopping …)	82.4%	7.0%	10.6%	23.694	<0.001
Having responsibilities toward siblings (taking care of homework, discussing with teachers …)	76.5%	10.3%	13.2%	30.053	<0.001
Help with administrative tasks (filling out forms …)	36.7%	37.8%	25.5%	56.829	<0.001
Providing financial support (ear money for family …)	9.8%	45.9%	44.3%	16.000	0.014
**Help provided to the person cared for**					
Help with mobility, bringing things	77.2%	7.4%	15.3%	21.374	0.002
Providing emotional support (listening to the relative, keeping them company …)	73.7%	11.4%	14.9%	29.682	<0.001
Being present to ensure everything is fine for the person cared for	68.2%	12.2%	19.6%	31.232	<0.001
Help the person cared for with daily activities (dressing, washing, eating …)	58.0%	14.6%	27.4%	16.594	0.011
Help with medical care (preparing medication, nursing …)	41.3%	25.1%	33.6%	13.455	0.036
Help with illness management (attending medical appointments, going to the pharmacy …)	32.4%	39.4%	28.2%	41.094	<0.001

**Table 2:** Perceived consequences of being a YC.

	Entire Sample			Comparison by Occupation	
	Often or Very Often	Never to Sometimes	Do Not Know	χ^2^	*p*
**General consequences**					
*Negative consequences*					
Sadness	81.2%	14.4%	4.4%	82.192	<0.001
Anxiety	66.2%	27.2%	6.6%	69.887	<0.001
Fatigue	65.0%	29.1%	5.9%	69.990	<0.001
Isolation	24.8%	63.5%	11.7%	27.053	<0.001
Physical health issues	20.8%	61.1%	19.1%	73.130	<0.001
Shame, guilt	19.7%	66.8%	13.5%	50.641	<0.001
Aggressivity	20.8%	68.0%	12.0%	56.126	<0.001
Dark thoughts, mutilation	10.9%	62.8%	26.3%	92.817	<0.001
Addictive behaviors	7.4%	56.3%	36.3%	140.068	<0.001
*Positive consequences*					
Maturity, responsibility	81.2%	14.4%	4.4%	13.970	0.030
Autonomy	72.3%	21.4%	6.3%	12.902	0.045
Empathy, altruism	56.8%	31.3%	11.9%	17.460	0.008
Personal enrichment	22.0%	55.9%	22.0%	59.408	<0.001
Self-confidence	20.8%	60.1%	19.1%	47.653	<0.001
**School consequences**					
Concentration issues	64.1%	31.1%	4.9%	59.929	<0.001
School difficulties	49.5%	44.7%	5.8%	81.312	<0.001
Absenteeism, lateness	48.9%	45.2%	4.9%	106.921	<0.001
Impact on educational progress	46.8%	42.2%	10.9%	76.972	<0.001
Repeating a class	24.6%	63.5%	11.9%	91.051	<0.001
Behavioral issues	24.3%	67.8%	8.0%	46.686	<0.001
School overinvestment	18.1%	62.5%	19.4%	58.182	<0.001
Stigmatization or bullying	6.2%	75.6%	18.2%	24.809	<0.001

**Table 3:** Suggestions to better identify and support YCs.

	Entire Sample			Comparison by Occupation	
	Relevant	Not Relevant	Do Not Know	χ^2^	*p*
**Enhance identification of young carers at school**					
Ensure a better follow-up for school change (primary, secondary, and high school)	96.4%	1.4%	2.3%	11.129	0.084 *
Share information between professionals at school	96.1%	1.5%	2.4%	40.514	<0.001 *
Collect information from the youth and the family	84.2%	7.3%	8.5%	10.364	0.110
**Propose school arrangements**					
Implementation of personalized tutoring or help with homework	88.0%	5.3%	6.7%	15.620	0.016
Adaptation of deadlines to provide personal work	76.9%	13.9%	9.2%	17.963	0.006
Establishment of committees to follow up with pupils/having a school career referent for young carers	69.9%	16.1%	14.0%	33.699	<0.001
Arrangements for time schedule	51.2%	34.5%	14.3%	18.702	0.005
Authorization to use a phone to contact the cared-for person during school time	36.9%	44.6%	18.4%	11.268	0.080
**Help and/or refer the youth and the family**					
Actively listening to the youth	98.7%	0.3%	1.0%	11.336	0.079 *
Encourage contact between the youth and social and healthcare professionals at school	95.2%	1.4%	3.3%	34.371	<0.001 *
Make contact with the family to discuss the situation	85.7%	4.9%	9.4%	41.885	<0.001
Referring the youth to professionals outside of school	85.2%	5.0%	9.8%	70.887	<0.001
Make contact with social services	66.0%	12.6%	21.4%	100.656	<0.001
**Raising awareness**					
Promote discussion times between youths about illness and disability	93.8%	2.1%	4.1%	22.266	0.001 *
Discourage stigmatization/bullying related to an illness or a disability in the family environment	92.7%	2.2%	5.1%	10.366	0.110 *
Make documents available in the school on illness, disability, and help provided within the family (flyer, website of the school, etc.)	84.2%	9.0%	6.8%	10.693	0.098

Note: χ^2^ with a * should be taken with caution as the theoretical frequency is less than 5.

## Data Availability

The datasets used and analyzed during the current study are available from the corresponding author upon reasonable request.
